# [Retracted] MicroRNA-18a enhances the radiosensitivity of cervical cancer cells by promoting radiation-induced apoptosis 

**DOI:** 10.3892/or.2025.9023

**Published:** 2025-11-17

**Authors:** Sha Liu, Xiaofen Pan, Qin Yang, Lu Wen, Yao Jiang, Yingchao Zhao, Guiling Li

Oncol Rep 33: 2853–2862, 2015; DOI: 10.3892/or.2015.3929

Following the publication of this paper, it was drawn to the Editor's attention by a concerned reader that the ‘Mock’ and ‘mimic-18a NC’ data panels shown in Fig. 5A for the SiHa flow cytometric (FCM) experiments were remarkably similar in appearance, even though their gated percentages were reported differently. Upon performing an independent analysis of the data in the Editorial Office, it came to light that various of the data quadrants shown in this figure were strikingly similar to FCM data in articles written by different authors at different research institutes that were published subsequently in other journals, one of which has been retracted on account of data re-use issues; in addition, data were apparently duplicated in Fig. 10A, where the same data had been used to show the results of differently performed experiments.

Given the nature of the contentious issues repported above, the Editor of *Oncology Reports* has decided that this paper should be retracted from the Journal on account of a lack of confidence in the presented data. The authors were asked for an explanation to account for these concerns, but the Editorial Office did not receive a reply. The Editor apologizes to the readership for any inconvenience caused.

